# Texting Lost-to-follow-up PrEP Patients from a San Francisco Sexual Health Clinic

**DOI:** 10.1007/s11121-022-01397-x

**Published:** 2022-07-16

**Authors:** Kelly A. Johnson, Montica Levy, Hannah Brosnan, Robert P. Kohn, Stephanie E. Cohen

**Affiliations:** 1 Division of Infectious Diseases, University of California San Francisco, 513 Parnassus Ave., Rm S380, San Francisco, CA 94143 USA; 2 Population Health Division, San Francisco Department of Public Health, San Francisco, CA USA; 3 Los Angeles County Department of Public Health, Los Angeles, CA USA

**Keywords:** PrEP, Retention, Persistence, Program evaluation, Public health, COVID-19

## Abstract

It is critical to understand what happens when PrEP patients are lost-to-follow-up (LTFU) and, where appropriate, attempt to re-engage them in care with the goal of preventing future human immunodeficiency virus (HIV) acquisition. We evaluated the benefits and limitations of using text-based outreach to re-engage with LTFU PrEP patients and offer re-initiation of PrEP care. Using text-messaging, we surveyed San Francisco City Clinic patients who started PrEP from January 2015 to October 2019 and were LTFU by October 1, 2020. Our goals were to better understand (1) whether our patients remained on PrEP through another provider or source, (2) why patients choose to discontinue PrEP, and (3) whether text-based outreach could successfully re-engage such patients in care. Multiple-choice survey questions were analyzed quantitatively to determine the proportion of respondents selecting each option; free-text responses were analyzed qualitatively using an inductive approach to identify any additional recurring themes. Of 846 eligible survey recipients, 130 responded (overall response rate 15.4%). Forty-two respondents (32.3%) were still on PrEP through another provider while 88 (67.7%) were not. Common reasons for stopping PrEP included: COVID-19–related changes in sex life (32.3% of responses), concerns regarding side effects (17.7%), and the need to take a daily pill (8.3%). Free text responses revealed additional concerns regarding risk compensation. While 32 participants agreed to be contacted by City clinic staff for PrEP counseling, only 6 were reached by phone and none of the six subsequently restarted PrEP. We learned that text messaging is a possible approach to survey certain PrEP program participants to determine who is truly LTFU and off PrEP, and to better understand reasons for PrEP discontinuation. While such information could prove valuable as programs seek to address barriers to PrEP retention, efforts to improve acceptability and increase response rates would be necessary. We were less successful in re-engaging LTFU patients in PrEP care, suggesting that text-messaging may not be the optimal strategy for this purpose.

## Introduction/Background

Pre-exposure prophylaxis (PrEP), typically with daily oral tenofovir disoproxil fumarate/emtricitabine (TDF/FTC), is a highly effective HIV prevention strategy (Fonner et al., 2016; Riddell et al., 2018) and a key component of the Ending the HIV Epidemic initiative in the USA (Giroir, 2020). Yet, sub-optimal long-term retention in PrEP care remains a primary challenge to the real-world effectiveness of PrEP (Serota et al., 2018). While PrEP retention rates were relatively high (ranging 69–92%) in clinical trials and demonstration projects, they have been estimated at just 15–62% in non-research settings (Wu et al., 2020). PrEP patients who are lost-to-follow-up (LFTU) are more likely to acquire HIV compared with those retained in care. This has been demonstrated among PrEP cohorts in Los Angeles (Shover et al., 2019) and in Montreal (Greenwald et al., 2019), which respectively observed HIV seroconversion rates of 2.1 and 3.9 per 100 person-years among people who discontinued PrEP versus 0.1 and 0/100 person-years among people who did not.

At San Francisco City Clinic (SFCC), San Francisco’s only municipal STI (sexually transmitted infection) clinic, PrEP has been offered as part of drop-in sexual health services since 2015. Despite a robust PrEP program providing PrEP to over 1000 patients per year — wherein PrEP counselors follow up with patients 2 weeks after PrEP initiation, before each quarterly PrEP visit, and at 2 weeks and 3 months after any missed quarterly visit — rates of PrEP retention-in-care have declined over time. From 2015 to 2019, 6-month retention in PrEP care at SFCC decreased significantly from 66 to 51% (*p*-trend < 0.05 using Cochrane-Armitage testing, SFCC unpublished data).

For PrEP programs to design and implement initiatives aimed to improve retention rates, it is critical to better understand the outcomes of individuals who are LTFU from clinic-based PrEP programs. Extrapolating from the HIV literature, for example, it is possible that not all individuals LTFU from a specific PrEP program are out of care and off anti-retroviral medications, as some will have transferred care elsewhere (Geng et al., 2013). It is also important to understand the reasons patients elect to discontinue PrEP — a topic that has been infrequently explored in prior studies of PrEP retention (Hojilla et al., 2018; Lankowski et al., 2019; Wu et al., 2020; Zucker et al., 2019). While — in accordance with the previously published prevention-effective adherence framework (Haberer et al., 2015) — some patients may appropriately opt to discontinue PrEP due to changes in sexual practices (such as entering a monogamous relationship) and/or the use of alternative HIV prevention strategies (such as condoms), others may be LTFU to PrEP care due to unaddressed concerns and/or barriers to accessing PrEP. For PrEP programs wanting to improve retention-in-care rates where appropriate for individual patients, it remains important to elicit, understand, and begin to address such concerns and barriers.

The purposes of this survey-based evaluation study were thus to (1) address whether SFCC LTFU PrEP patients remained on PrEP through a different provider and/or in a different location, (2) compare demographic characteristics of LTFU patients who were still taking versus who had truly discontinued PrEP, and (3) ascertain reasons for PrEP discontinuation. An additional primary goal was to evaluate the potential benefits and limitations of using text-message-based outreach to engage with LTFU patients and potentially reconnect them to PrEP care within our program.

## Methods

We used a text-message (short messaging service/SMS) based platform to survey SFCC patients who initiated PrEP from January 2015 to October 2019 and were LTFU (i.e., not seen in the clinic for a PrEP visit in over 6 months) as of October 1, 2020. Patients who would have otherwise been eligible but do not speak English (*n* = 66) and/or are without a working telephone number in the electronic medical records system (*n* = 27, two of whom also did not speak English) were excluded from survey participation. Patients who had previously opted out of receiving text messages from SFCC (*n* = 6) were also excluded. The survey was initially sent to all eligible patients in October of 2020 and resent twice throughout the month to patients who had previously not responded.

We leveraged Upland Mobile Commons as the platform to create and administer our survey. The survey questions (Appendix) were created within Mobile Commons using a coding language (liquid coding), then administered to eligible City Clinic patients who had previously agreed to receive text messages from our clinic (using an opt-out approach). Within the text-based survey, participants were first asked whether they were willing to take a survey about why people choose to start or stop PrEP. Participants could elect not to participate in the survey by answering “no” or simply ignoring this message. In the interest of protecting our patients’ privacy, the initial survey question did not explicitly identify individual survey recipients as having ever previously received services at SFCC.

The text-based survey (Appendix) consisted of a series of multiple choice and free text questions addressing whether LTFU SFCC patients remained on PrEP through an alternative clinic/source or had specific reasons for stopping PrEP. The survey was coded to use logic-based automated responses to gather additional data as appropriate based on each respondent’s answers to previous questions.

Responses to multiple-choice survey responses were then analyzed quantitatively, to identify the proportion of survey respondents selecting specific multiple-choice options. Free-text responses were analyzed qualitatively, using an inductive approach wherein a single coder reviewed all free text responses to explore any common recurring themes that were not previously presented in multiple choice options as to why patients may have opted to discontinue PrEP.

Phone numbers of survey respondents were matched to phone numbers listed for individual patients within our SFCC electronic medical record (EMR) to abstract clinical/demographic variables including patient age, race/ethnicity, self-reported number of sex partners in the last 3 months (as of the patient’s last SFCC visit), and any diagnosis of syphilis of any stage or chlamydia/gonorrhea in the last 12 months (again as of the patient’s last SFCC visit). In cases where a single phone number was listed for more than one PrEP patient (as when two PrEP patients elect to share one phone number), we excluded that phone number from analyses of sociodemographic/clinical variables, since we could not be certain which patient had provided a survey response. The match was conducted by an epidemiologist from the SFCC STD program and all patient identifiers were excluded from the dataset used for analysis. We then compared clinical/demographic characteristics of (A) survey respondents vs. non-respondents and (B) among survey respondents, those still taking vs. not taking PrEP. Categorical variables were compared using chi squared or Fisher’s exact testing; numerical variables were compared using Wilcoxon-rank sum. All statistical analyses were performed using STATA version 16.0.

Finally, within the survey, respondents not currently taking PrEP were offered the opportunity to discuss their concerns with an SFCC staff member, with the goal of potentially re-engaging LTFU patients in PrEP care. We tracked the number of survey respondents who (1) agreed to be contacted by SFCC staff through our text-based survey, (2) were successfully reached by SFCC staff for phone counseling, and (3) ultimately came to SFCC to restart PrEP with follow-up through March 2021.

## Results

Of the 846 eligible SFCC LTFU PrEP patients who received the text-based survey, 133 agreed to participate. Three respondents did not answer any additional questions beyond question #1, and were thus excluded from further analysis, giving an overall response rate of 15.4% (130/846).

### Survey Responses

**Fig. 1:**
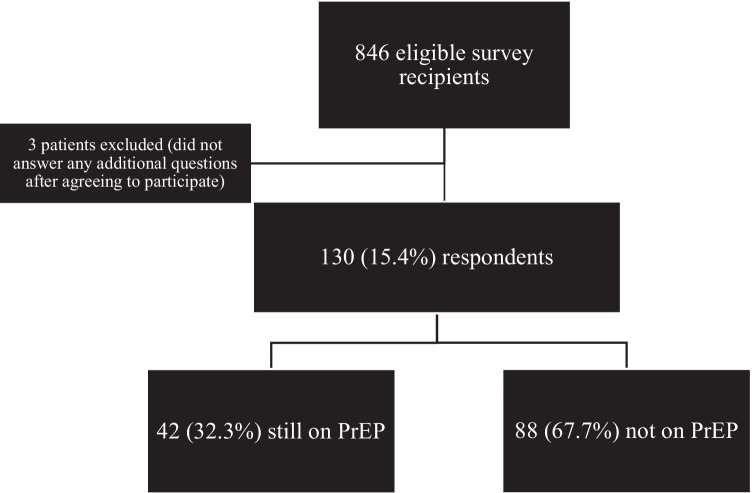
Participant flow diagram – Texting San Francisco City Clinic patients who initiated PrEP between January 2015 and October 2019 and were subsequently lost-to-follow-up (not seen in clinic in over 6 months) as of October 1, 2020

Among the 130 respondents, 42 (32.3%) were still on PrEP while 88 (67.7%) were not (Fig. 1). Ten of the 42 patients still on PrEP (24%) had moved away and were no longer living in the San Francisco Bay Area. Commonly listed alternative sources of PrEP outside of SFCC were primary care providers, especially those affiliated with a large healthcare maintenance organization and private insurance company (Kaiser Permanente, listed by 12/42 or 29% of survey respondents still on PrEP).

Among the 88 survey respondents no longer taking PrEP, common reasons for PrEP discontinuation selected among multiple choice options (with respondents able to select more than one option) included COVID-19–related changes in sex life (32.3% of responses); concerns regarding side effects (17.7% of responses); and the need to take a daily pill (8.3% of responses). Cost or having lost/changed insurance were less commonly selected options, at 5.2% and 4.2% of responses, respectively. Other, even less commonly selected reasons for PrEP discontinuation were perceived low risk of HIV acquisition (3.1%), having never started PrEP (3.1%), hearing negative messaging about PrEP on social media (2.1%), a preference for condoms (2.1%), and difficulties coming into clinic for PrEP visits (1.0%).

Free text responses highlighted additional concerns regarding risk compensation among patients who stopped taking PrEP, with one participant noting: “When [I] was taking prep, [I] was more inclined to have unprotected sex with people [I] was not comfortable with. Now that [I] don’t take it, [I] have more incentive to resist impulses.” Two other participants similarly wrote: “I was making riskier choices [on PrEP], which led to other STIs” and “[I] don’t want to have multiple sex partners and if I took PrEP, [I] would be more encouraged to have sex which is not something I want to be engaging in.”

### Comparing Survey Respondents vs. Non-respondents and Participants Still on PrEP vs. No Longer Taking PrEP

Overall, of the 846 eligible SFCC patients who received our survey, 815 (96.3%) had phone numbers that were able to be linked to a unique patient’s chart in the SFCC electronic medical record (127/130 respondents and 688/716 non-respondents). Respondents were older than non-respondents (median 33 vs. 30 years; *p* < 0.001). A higher proportion of respondents were white compared with survey non-respondents (48.8% vs 38.7%; *p* = 0.055), though this trend did not demonstrate a statistically significant difference between groups. There were also no statistically significant differences between respondents and non-respondents in terms of number of reported sexual partners in the last 3 months or diagnoses of syphilis, chlamydia, or gonorrhea within the 12 months preceding their last SFCC visit (all with *p* > 0.05) (Table 1).

**Table 1:** SFCC PrEP LTFU Survey—Comparing Survey Respondents to Non-Respondents (total n = 815 unique patients available for analysis)

	**Non-respondents** **(*n* = 688)**	**Respondents** **(*n* = 127)**	Test of significance^*^
Median age (years)	30 (IQR 26, 37)	33 (IQR 28, 42)	*p* < 0.001
Race/Ethnicity			*p* = 0.055
Black	87 (12.6%)	11 (8.7%)	
Asian	155 (22.5%)	18 (14.2%)	
Hispanic	156 (22.7%)	31 (24.4%)	
Native American/PI	6 (0.9%)	3 (2.4%)	
Other/unknown/refused	18 (2.6%)	2 (1.6%)	
White	266 (38.7%)	62 (48.8%)	
Sexual partners in last 3 months (Median number)	3 (IQR 2, 6)	4 (IQR 2, 7)	*p* = 0.100
Syphilis of any stage in last 12 Months	66 (9.6%)	6 (4.7%)	*p* = 0.076
Gonorrhea/Chlamydia in last 12 months	272 (39.5%)	50 (39.4%)	*p* = 0.97

*SFCC* San Francisco City Clinic, *PrEP* pre-exposure prophylaxis, *LTFU* lost to follow-up (not seen for a PrEP visit at SFCC in the last 6 months as of October 1, 2020)

^*^Categorical variables were compared using chi squared or Fisher’s exact testing; numerical variables were compared using Wilcoxon-rank sum

In comparing survey respondents currently on PrEP vs. not on PrEP (Table 2), the only statistically significant difference was that those still taking PrEP had reported more sexual partners in the 3 months leading up to their last SFCC clinic visit, with a median 5 [interquartile range (IQR) 3–10] vs. 3 (IQR 2–6) partners (*p* = 0.015). There were no other statistically significant differences between respondents currently on PrEP vs. not on PrEP in terms of age, race/ethnicity, or diagnoses of bacterial STIs within the 12 months prior to their last SFCC visit (all with *p* > 0.05).

**Table 2:** SFCC PrEP LTFU survey: comparing patients still on PrEP vs. patients who discontinued (total *n* = 127 unique patients available for analysis)

	**Not on PrEP** **(*n* = 85)**	**Still on PrEP** **(*n* = 42)**	Test of significance^*^
Median age (years)	33 (IQR 28, 42)	32 (IQR 28, 42)	*p* = 0.59
Race/ethnicity			*p* = 0.32
Black	5 (6%)	6 (14%)	
Asian	11 (13%)	7 (17%)	
Hispanic	23 (27%)	8 (19%)	
Native American/PI	1 (1%)	2 (5%)	
Other/unknown/refused	2 (2%)	0 (0%)	
White	43 (51%)	19 (45%)	
Sexual partners in last 3 months (Median number)	3 (IQR 2, 6)	5 (IQR 3, 10)	*p* = 0.015
Syphilis of any stage in last 12 months	3 (4%)	3 (7%)	*p* = 0.37
Gonorrhea/chlamydia in last 12 months	31 (36%)	19 (45%)	*p* = 0.34

*SFCC* San Francisco City Clinic, *PrEP* pre-exposure prophylaxis, *LTFU* lost to follow-up (not seen for a PrEP visit at SFCC in the last 6 months as of October 1, 2020)

^*^Categorical variables were compared using chi squared or Fisher’s exact testing; numerical variables were compared using Wilcoxon-rank sum

### Efforts to Re-engage LTFU PrEP Patients Via Text Messaging

Within the survey, respondents who reported not currently taking PrEP were offered the opportunity to discuss their concerns with an SFCC staff member. Thirty-two (36.4%) of the 88 participants not currently on PrEP agreed to be contacted by SFCC staff for PrEP counseling, of whom 30 were called by phone. Six of the 30 were successfully reached by phone, of whom only one came to clinic to restart PrEP but left without being seen. Of the remaining survey respondents who requested contact from an SFCC staff member but were subsequently unreachable by phone, two later came to SFCC and ultimately received a PrEP prescription. It is unknown whether the LFTU survey played a role in their decision to re-initiate PrEP.

## Discussion/Conclusions

This analysis demonstrates that it is possible to engage at least a subset of LTFU PrEP patients by text to elicit their current PrEP status as well as their concerns regarding PrEP use. We learned that one third of LTFU SFCC patients had not actually stopped PrEP, instead transferring their PrEP care elsewhere, and that the top three most commonly reported reasons for PrEP discontinuation included COVID-19–related changes in sexual practices, concerns around side effects, and/or the need to take a daily pill. That said, survey response rates were overall low — and we were less successful in re-engaging LTFU patients in care — suggesting that there are opportunities for improvement and lessons to share with public health PrEP programs considering text-based outreach.

Importantly, and unique from prior studies of PrEP retention, we were able to assess outcomes as to whether patients enrolled in an STI-clinic–based PrEP program truly stopped taking PrEP after being LTFU from the program. The results suggest that a LTFU status at a particular PrEP program does not necessarily imply PrEP discontinuation, as one third of survey respondents (ten of whom had moved away) had instead transferred their PrEP care to another provider and continued on prophylactic therapy.

Among respondents who discontinued PrEP, this analysis offers new insights as to why patients may choose to do so. First, reflecting the fact that this survey was administered in October 2020— when much of the U.S. remained under COVID-19 related “shelter-in-place” or “stay at home” orders — COVID-19–related changes in sex life was the most frequently selected reason for PrEP discontinuation. This is consistent with previously reported studies, with an international review of 20 articles from 12 countries confirming that many people (40–75%) reduced their number of sexual partners during the COVID-19 era (Renfro, 2020). Perhaps in part for this reason, coupled with COVID-19–related disruptions in sexual health services, modeling studies estimate that there was a 21% decrease in overall PrEP prescriptions and a 28% decrease in new U.S. PrEP users from March to September of 2020 when compared with the same time period during the prior year (Huang et al., 2021). Future evaluation research will be needed to explore whether and how patients who stopped PrEP during the COVID-19 era — including those LTFU at SFCC — may re-engage in PrEP care as the COVID-19 pandemic resolves or evolves over time.

Other, less frequently cited reasons for stopping PrEP identified in this LTFU survey included concerns around insurance/cost, side effects, and the need to take a daily pill. Such concerns around PrEP cost (Arnold et al., 2017; Ezennia et al., 2019; Whitfield et al., 2018), perceived or actual adverse effects (Arnold et al., 2017; Ezennia et al., 2019; Lankowski et al., 2019; Whitfield et al., 2018), and need for daily dosing (Whitfield et al., 2018) have been similarly identified in prior PrEP retention studies. Such concerns are important to elicit from PrEP patients, as they can potentially be addressed with interventions such as enrollment in patient assistance programs (with the goal of minimizing any PrEP-related financial burdens) or changes in PrEP dosing (from daily to 2-1-1 or long-acting injectable PrEP), to address concerns related to daily dosing or taking oral medications (Molina et al., 2015).

A small number of survey respondents (3.1% of responses) indicated that they had stopped PrEP due to perceived low risk of HIV acquisition. Objectively, when comparing survey respondents still taking PrEP to those who had stopped, respondents who reported higher numbers of sexual partners at their last SFCC visit were statistically more likely to still be on PrEP, suggesting that PrEP continuation may indeed be correlated with risk of HIV exposure/infection. This finding is consistent with prior data from Maryland, wherein patients reporting multiple sexual partners (3+ versus 0–2 partners) had a decreased risk of stopping PrEP (Zucker et al., 2019). Yet — while some patients, in accordance with the prevention-effective adherence framework, may appropriately choose to discontinue PrEP during periods of minimal or no risk of HIV exposure (Haberer et al., 2015) — SFCC patients who had discontinued PrEP still reported multiple sexual partners at their last clinic visit before PrEP discontinuation, and thus could have ongoing indications for PrEP despite having discontinued prophylaxis. Further research is needed to better understand how to communicate with patients regarding their individual risk of HIV acquisition and potential reasons to continue PrEP when appropriate, even as sexual practices change over time.

A final reason cited for PrEP cessation within the LTFU survey involved concerns that PrEP led to an increase in sexual activity. Whether PrEP is associated with changes in sexual practices on a population level is controversial. On the one hand, analyses of PrEP studies, including randomized clinical trials, open label extensions, and demonstration projects, have demonstrated no differences or changes in self-reported sexual behaviors among participants taking and not taking PrEP (Fonner et al., 2016; Grant et al., 2010; Marcus et al., 2013; Montaño et al., 2019). On the other hand, real-world experience with U.S. PrEP cohorts in Seattle (Montaño et al., 2019), San Francisco (Volk et al., 2015), Providence, Rhode Island (Oldenburg et al., 2018), Chicago (Newcomb et al., 2018), and elsewhere indicates that some people report increased numbers of condomless sex partners or decreased condom use — and may be diagnosed with more bacterial STIs (Traeger et al., 2018) — while on PrEP. In this analysis, some respondents indicated that they discontinued PrEP due to a self-perception that taking PrEP led to unwanted changes in their sexual practices. Checking in with patients about their sexual practices and offering culturally informed, non-judgmental information about ways to protect oneself from non-HIV STIs (e.g., condoms, frequent STI screening, and partner notification and treatment for bacterial STIs), may help support patients who have ambivalence about whether to continue PrEP.

Unlike prior studies (Chan et al., 2019; Zucker et al., 2019), race/ethnicity and younger age were not associated with PrEP discontinuation in this LTFU analysis. The survey also did not explicitly identify barriers such as: (1) HIV and/or gender-related stigma; (2) medical mistrust; (3) structural challenges like unstable housing or unreliable transportation; (4) limited or no access to healthcare; (5) lack of social support; or (6) mental health and/or substance use disorders as reasons for stopping PrEP. These concerns, however, have been raised in prior PrEP retention studies (Chan et al., 2019; Ezennia et al., 2019; Wu et al., 2020) and remain important topics of exploration for future studies on PrEP persistence versus discontinuation.

A primary limitation of this evaluation included a low survey response rate. Self-selection bias, wherein people who maintained some level of interest in PrEP may have been more likely to respond to the survey, could have resulted in a sample under-representative of patients who had entirely lost interest in PrEP and/or had major reservations regarding PrEP use. With this initial version of our survey, we were also unable to reach individuals who did not have a working telephone number or who did not speak English, meaning that those with marginal housing and/or limited English proficiency were under-represented. We were similarly unable to reach any patients who had previously opted out of receiving text messages from our clinic. We did not offer incentives for survey participation, and did not use strategies such as flyers, posters, or in-person communications to raise patient awareness of this survey ahead of time. To improve response rates, programs considering similar outreach to LTFU PrEP patients could consider developing promotional materials, offering incentives, re-creating the survey in multiple languages, and/or offering the survey in more than one format (text, e-mail, paper, phone conversations, etc.). Programs considering text-based outreach to LTFU PrEP patients could also consider assessing the acceptability and feasibility of implementing such an outreach program from the perspective of clinic staff members, something that was outside the scope of this project.

Due to our lower response rates, we did not have sufficient power to stratify our analyses by particular clinical or sociodemographic characteristics. Additionally, within the overall low survey response rates, people of color had even lower response rates — a finding of particular concern, given that the U.S. HIV epidemic disproportionately impacts communities of color (Centers for Disease Control and Prevention (CDC), 2021), and that prior studies have found PrEP uptake and persistence rates to be consistently lower among Black individuals compared with other races (Chan et al., 2019; Ezennia et al., 2019; Zucker et al., 2019). Further efforts (potentially such as focus groups or key informant interviews) would be needed to determine whether and how to adapt our LTFU survey to improve its acceptability and response rates, particularly among people of color.

Finally, while this survey was successful in eliciting certain LTFU PrEP patients’ current PrEP status, as well as potential concerns related to PrEP, we were less successful in re-engaging LTFU patients in PrEP care through text messaging. Only one survey recipient who asked to be contacted by SFCC staff within the survey platform ultimately came to clinic to restart PrEP, and unfortunately left before receiving a PrEP prescription. This suggests that text-based outreach may not be the ideal strategy for re-engaging LTFU PrEP patients in care in our clinical setting and/or that alternative outreach strategies (potentially such as phone calls, peer counseling, social media ads, television commercials, etc.) should perhaps be considered instead.

## Conclusions

In summary, we found that text messaging is a possible approach to survey certain PrEP program participants to determine who is LTFU and truly off PrEP, and to better understand reasons for PrEP discontinuation. Such information could prove invaluable as programs seek to develop new methods of PrEP care and delivery that could address barriers to ongoing PrEP engagement. Our survey had a low response rate and was not successful in re-engaging LTFU PrEP patients in PrEP, suggesting that text messaging may not be the optimal strategy for this purpose, and that additional approaches for surveying LTFU PrEP patients and supporting retention and engagement in PrEP care are needed.

## Data Availability

The primary data discussed within this study is available for review upon request.

## Appendix. Text-message Survey Distributed to San Francisco City Clinic Patients who Initiated PrEP Between January 2015 and October 2019 and were Subsequently Lost-to-follow-up (not Seen in clinic in Over 6 Months as of October 1, 2020)

1. **Hi! We at San Francisco City Clinic are hoping to learn more about why people choose to start or stop PrEP! Are you willing to take our quick survey? Text Yes or No.**
  - If No ➔ Thank you. Remember you can always visit our website at https://www.sfcityclinic.org/ or call us at 415-487-5537 to learn more about PrEP.
  - If Yes ➔ Great! Let's continue!
2. **Are you still living in the Bay Area? Text Yes or No.**
3. **Are you currently taking PrEP? Text Yes or No.**
  - If yes ➔ Great! Glad PrEP is still working out for you. Where do you get your PrEP? Please answer this question, then text STOP to end this survey.
  - If no ➔
4. **Why are you not taking PrEP now? Reply with the LETTER next to the reason you choose.**
  - Prefer condoms
  - Concerned about cost
  - Concerned about side effects
  - Don't want to take a pill every day
  - Coming into clinic was too hard
  - Lost/changed insurance
  - Heard bad things about PrEP on internet/social media
  - Did not feel I was likely to get HIV
  - I never started PrEP
  - My sex life changed because of COVID-19
  - Other
    - If K (not on PrEP now): Please share your reason for not taking PrEP. (Free text)
    - If I (never started PrEP): Why didn't you start PrEP? (Free text)
    - If any other answer: Is there anything else we should know about why you aren't on PrEP? (Free text)
5. **We would love to address any concerns you might have about PrEP. You may not know that there is a new option for PrEP that doesn't involve a pill every day and that works for some patients, and that we can help you get PrEP for free! Would you like us to contact you to talk more about PrEP? Text Yes or No.**
  - If yes ➔ Glad you are interested in learning more about PrEP! **We will call you in the next few days.** You can always also call us at 415-487-5537.
  - If no ➔ Thank you. Remember you can always visit our website at https://www.sfcityclinic.org/ or call us at 415-487-5537 to learn more about how you can access PrEP.

## Code Availability

All statistical coding for this evaluation was performed using STATA version 16.0. This code is available for review upon request.
